# Current News and Opinion

**Published:** 1884-03

**Authors:** 


### Current News and Opinion.

### MICHIGAN STATE DENTAL SOCIETY.

The annual meeting of the Michigan State Dental Society will be held in the city of Detroit, commencing the last Wednesday in March, 1884, and continuing two days. President, Dr. W. H. Dorrance, Ann Arbor; Secretary, Dr. J. B. McGregor, Port Huron.

The following papers will be read:

| | |
|---|---|
| Prosthetic Dentistry, - - | Dr. Dorrance. |
| Atmospheric Dentures, - - | Dr. Land. |
| Diseases of the Gums, - - | Dr. Cleland. |
| Oral Surgery, - - - - | Dr. Case. |
| Histology, - - - - | Dr. Britton. |
| Methods and Materials for Filling Teeth | Dr. Moore. |
| Treatment of Deciduous Teeth - | Drs. Taft and Cowie. |
| Pulpless Teeth, - - - | Dr. Douglas. |
| Exposed Pulps, - - - | Dr. Rowley. |
| Dental Caries - - - - | Dr. Shattuck. |

A cordial invitation is extended to all practicing dentists to be present.

### VULCANIZING INDIA RUBBER.

Accidents have frequently occurred, especially in dental workshops, from the use of too high a temperature in melting and vulcanizing India rubber. Moreover, complicated apparatus is required for vulcanizing by dry heat. According to the *Moniteur Produits Chimiques*, this apparatus can be replaced by a bath of any liquid boiling at 140° or 150° C. (285° to 300° F.), at which temperature the sulphur unites with the rubber.

The cheapest salt for such a bath is chloride of calcium; but other solutions, such as acetate of soda and carbonate of potash, can be employed; also glycerine, oils, and paraffine. These liquids can be employed in ordinary metallic vessels. Of course, the India rubber and sulphur solution must be in an air-tight vessel, as before.—*Scientific American.*

### STATE OF THE GUMS IN PREGNANCY.

M. Delestre has observed that not only in pregnancy, but during the menstrual periods, the gums in the female are congested, swollen and softened. The gingival troubles commence about the second month of pregnancy. Didsbury describes three degrees of gingivitis of pregnancy. In the third the gums are so inflamed that they have a reddish violet color, are swollen, and the interdental portion is clearly shown. Tartar and epithelial debris accumulate around the teeth. This inflammation may extend to the alveolar-dental periosteum, for the teeth seem to lose their lime, become elevated and may fall out. This gingivitis is situated particularly in the anterior portion of the jaws; it rarely goes back of the canine teeth. Only the convex surface of the jaws is attacked. The treatment should be energetic; the tartar should be removed and the inflammation treated by stringent preparations, chlorate of potash, etc., and in grave cases with tincture of iodine, chromic acid, and hydrate of chloral mixed with some astringent tincture.—*Jour. de Med. de Paris.*—*Med. News.*

---

### "CAN'T AFFORD IT."

Such was the reply of a professional brother recently, when asked to subscribe for a dental magazine. Two dollars and fifty cents a year for a literary periodical. What a heavy tax! Hardly five-sevenths of a cent a day, to be sure, yet a monstrous sum of money to pay for reading matter.

To become the happy possessor of three of the leading dental journals published would cost about two cents a day; and who does not daily squander far more than that amount in divers ways?

Many members of our calling who imagine that they cannot afford to provide themselves with literary matter, somehow manage to procure sufficient means to lay in a stock of good cigars, attend places of amusement, and expend lavishly for other indulgences, a tithe of which would supply them with a goodly array of journals, from which they might derive much real, wholesome, practical benefit.

Think of this, ye who declare that you "cannot afford it."

C. E. F.

### MISSISSIPPI VALLEY DENTAL SOCIETY.

The fourteenth annual meeting of this society will be held at the Ohio Dental College in Cincinnati, commencing Wednesday, March 5th, 1884, at ten o'clock A. M. The executive committee have presented the following programme:

1. Artificial crowns upon roots; best method of adapting them.
2. What means are best adapted for maintaining the teeth and mouth in a healthy condition?
3. What progress has been made in arresting and preventing decay of the teeth in the last five years?
4. In what respect, if any, are decayed teeth better filled now than ten years ago?
5. What valuable medicinal agents have been introduced into dental practice in the last three years? For what purpose is each one used?
6. What improvements, if any, have been made in artificial dentures during the last five years; and what further improvements are desirable?

---

### HE KNEW IT.

Dr. Miller * * * advocates the chemical theory of decay, as is shown by his published papers and by the letters of Prof. Wright in the January number of our journal. Of course, we expected nothing else, as we know that Dr. Miller was repeating substantially the same experiments we had tried between 1855 and 1862.—*Editorial in Ohio State Journal.*

What *will* this world do when Dr. Watt dies? It might have wabbled on for a while, had it not been for the more than Alexandrian holocaust, that most unfortunate fire that destroyed the records of all the really original experiments that this world is likely to know anything of for some time to come.

---

### CHANGE OF LOCATION.

Dr. H. V. Wollison, formerly of Port Jervis, N. Y., a graduate of the New York College of Dentistry, has removed to London, and is now associated with Dr. J. J. Wedgwood, 15 George Street, Hanover Square, West.

### COMPLIMENTARY.

We have no more of journalistic conceit than the next man, but we cannot resist the temptation to give place to the following very complimentary notice from that sterling journal, THE VIRGINIA MEDICAL MONTHLY, published at Richmond, Va.:—*Editor.*

"THE INDEPENDENT PRACTITIONER."—We feel that we must speak a word of praise for this most excellent dental journal. Although of course our practice lies entirely outside of the domain of dentistry, there is scarcely one of our exchanges more carefully perused; and we take special occasion to mention the January number of the journal. The leading article by the American dentist, Dr. N. S. Jenkins, of Dresden, Germany, entitled "A Day's Practice," is one of the most readable professional papers we have met with for some time. We wish the editor, Dr. Barrett, of Buffalo, all the success he so well deserves.

---

### DENTAL COLLEGE IN FRANCE.

For many years the standard of Dental Education in France has been at a low ebb, but the prospectus of a new Dental College, to be located in Paris, is now out, and from it we judge that a new departure is to be taken. *The Institut Odontotechnique de France* proposes to give to French dental students all the advantages offered in the American colleges and the English hospitals. Dr. E. A. Bogue, of New York and Paris, will occupy the chair of Operative Dentistry.

---

### TEXAS DENTAL ASSOCIATION.

The annual meeting of the Texas Dental Association will be held in San Antonio on the last Tuesday in April. The programme has not yet been issued. We acknowledge the courtesy of an earnest invitation to be present.

---

### NEW USE FOR ELECTRICITY.

A very convenient electric lighter is being sold throughout the country for only five dollars.

### ANÆSTHETICS FROM A MEDICO-LEGAL POINT OF VIEW.

Dr. J. G. Johnson, of Brooklyn, comes to the following conclusions in the *Annals of Anatomy and Surgery:*

Anæsthetics do stimulate the sexual functions, the ano-genital region being the last to give up its sensitiveness. Charges made by females under the influence of an anæsthetic should be received as the testimony of an insane person is. It cannot be rejected; but the *corpus delicti aliunde* rule should be insisted on. Dentists or surgeons who do not protect themselves by having a third person present do not merit much sympathy.

Gross violations of the well-known rules of administering anæsthetics, life being lost thereby, will subject the violator to a trial on a charge of manslaughter.

A surgeon allowing an untrained medical student to administer anæsthetics, life being lost thereby, will subject the surgeon himself to a suit for damages. What he does through his agent he does himself.

The physician who administers an anæsthetic should attend to that part of the business and nothing else. He should have examined the heart and lungs beforehand. He should have the patient in the reclining position, with his clothes loose, so as not to interfere with respiration; should have his rattooth forceps, nitrate of amyl, and ammonia, and know their uses, and when to use them, and how to perform artificial respiration.—*Quarterly Epitome.*

---

### A CORRECTION.

Last month, in an editorial notice of the dissolution of the firm of F. W. Leonard & Co., we perhaps left the impression in the minds of our readers that Mr. Leonard retires from business altogether. This is not the case, as will be seen by a reference to his new advertisement in this number.

---

### ODONTOLOGICAL SOCIETY OF GREAT BRITAIN.

Dr. W. St. Geo. Elliott, of London, has been elected resident member of the Council of the British Odontological Society.

