# An Error

**Published:** 1884-04

**Authors:** 


### AN ERROR.

We were mistaken when, in the March number, we said that Dr. E. A. Bogue had accepted the chair of Operative Dentistry in the *Institut Odontotechnique de France.* He has only consented to act as one of the Clinical Demonstrators. We gave the item of the authority of an English journal.

